# Critical thinking disposition in Chinese students: a meta-analysis of studies published from 2000 to 2025

**DOI:** 10.3389/fpsyg.2026.1673165

**Published:** 2026-02-23

**Authors:** Shengli Guo, Huiyong Fan, Jianzhong Xu

**Affiliations:** 1 College of Education, Bohai University, Jinzhou, China; 2 Center of Psychological Research and Education, Bohai University, Jinzhou, China; 3 Department of Counseling Higher Education Leadership Educational Psychology and Foundations, Mississippi State University, Starkville, MS, United States

**Keywords:** Chinese students, critical thinking disposition, effect of university type, grade effect, inventory, major effect, meta-analysis

## Abstract

The level of Chinese students’ critical thinking disposition has been widely debated for the past three decades, yet previous studies have reported inconsistent findings and lacked quantitative synthesis. To address this gap and clarify the current state of critical thinking dispositions among Chinese students, the present study conducted a meta-analysis of 491 primary studies, encompassing 688 independent samples and 146,625 participants. The analysis found that the averaged scores of Chinese students’ critical thinking disposition fluctuated between 3.684 and 3.909 on a six-point Likert scale. Moderator analyses revealed that grade level, major type, publication type, and university type significantly influenced effect sizes, whereas the methodological quality of the primary studies was not significantly associated with effect sizes. As the first global study to provide a precise estimate of Chinese students’ critical thinking disposition, it highlights key areas for further research and discusses relevant theoretical and educational implications.

## 1 Introduction

### 1.1 Background

Recently the status of Chinese students’ critical thinking has intrigued the international research community (Fan and See, 2022; Jiang et al., 2024; Tian and Low, 2011; Zhang, 2017).

Critical thinking is a complex, multi-component construct (please refer to Abrami et al., 2008, 2015 for detailed discussions on the definition of critical thinking; Fan and See, 2022; Liu et al., 2026). Although perspectives on its internal structure vary, scholars generally identify four key elements: skills, disposition, style, and ethical dimension (Baker et al., 2021; Facione, 1990; Fan and See, 2022; Xie et al., 2025).

Critical thinking skills are defined as the cognitive capacity for critical reasoning, encompassing analysis, evaluation, and inference (Facione, 1990; Facione et al., 2000; Fan and See, 2022; Huber and Kuncel, 2016; Pithers and Soden, 2000; Barta et al., 2022). Dispositions represent the affective aspect of critical thinking, reflecting an individual’s internal inclination toward decision-making and actions. They include several key dimensions such as open-mindedness, truth-seeking (Facione,1990; Facione et al., 2000; Fan and See, 2022; Barta et al., 2022). The critical thinking style refers to the overall approach to applying critical thinking in problem solving, incorporating dimensions such as engagement and information seeking (Fan and See, 2022; Lamm and Irani, 2011).

The ethical dimension is related to the students’ awareness of social hierarchical structures and ultimately cultivates the ability to consider broader perspectives and moral consequences (Xie et al., 2025). This dimension has not been considered by the existent measuring tools. The Chinese educational discourse particularly emphasizes the ethical dimension‌ of critical thinking (Xie et al., 2025).

Among the four components, critical thinking dispositions function as the motivational driver, as valuing and utilizing critical thinking encourage individuals to develop critical skills and ultimately determines whether critical thinking is applied in the complex problem-solving situation (Abrami et al., 2008, 2015; Facione et al., 2000; Fan and See, 2022; Liu et al., 2026). Because dispositions determine whether critical thinking skills are activated and applied, they are theoretically regarded as the foundational element shaping actual critical thinking behavior. Therefore, critical thinking dispositions are the most crucial component and warrant systemic study. The current paper will focus on the dispositions of critical thinking and estimate the true score of Chinese students’ critical thinking dispositions.

The debate over the critical thinking disposition of Chinese students has persisted for the past three decades. Earlier studies were based on personal observations or investigative methods (Atkinson, 1997; Tian and Low, 2011; Paton, 2005; Shaheen, 2016; Fan and See, 2022; Jiang et al., 2024). As more studies accumulated, inconsistencies in the findings began to surface.

Although previous work has repeatedly highlighted the importance of understanding Chinese students’ critical thinking, existing evidence remains fragmented, inconsistent, and methodologically limited (Dennett and DeDonno, 2021; Fan and See, 2022; Jiang et al., 2024). This gap prevents researchers from drawing theoretically grounded or empirically reliable conclusions about the actual level of Chinese students’ critical thinking dispositions.

To date, there is insufficient systematically organized evidence to estimate the true scores of Chinese students’ critical thinking disposition, and to determine whether Chinese students outperform or underperform compared to their international counterparts. Fan and See (2022) identified three major reasons for this uncertainty. First, the number of studies using standardized critical thinking measurement is limited. Second, few studies have conducted quantitative comparison between Chinese students and their international peers. Third, the studies reviewed by Fan and See (2022) focused exclusively on college students, limiting the generalizability of the findings to other populations.

### 1.2 The purpose and research questions of the current paper

The purpose of this study is to estimate the actual state of Chinese students’ critical thinking disposition and identify the study features that contribute to the inconsistency found in the primary studies included. Specifically, the research questions for this study are as follows.

*Question 1*: What is the real status of critical thinking disposition among Chinese population students?

*Question 2*: Does the hometown location moderate the difference in the critical thinking disposition scores?

*Question 3*: Does grade level significantly moderate critical thinking disposition?

*Question 4*: Does major type significantly moderate critical thinking disposition scores?

*Question 5*: Does university type significantly moderate critical thinking disposition?

*Question 6*: Is the moderating effect of publication type significant?

## 2 Methods

To quantify the critical thinking disposition of Chinese students and analyze potential influencing factors, this study employed a meta-analytic approach. Meta-analysis is a systematic quantitative research synthesis method that aims to integrate results from multiple independent primary studies and estimate overall effects based on standardized effect size metrics (Nakagawa et al., 2023). This method allows the integration of research data across different educational stages, disciplines, and regions, providing statistical support for drawing representative conclusions.

### 2.1 California critical thinking disposition inventory (CCTDI)

#### 2.1.1 Three versions of CCTDI

##### 2.1.1.1 English version of CCTDI

The English version of CCTDI has 75 items across seven dimensions: Truth-seeking, Open-mindedness, Analyticity, Systematicity, Self-confidence, Inquisitiveness and Cognition Maturity (Facione et al., 1994, 2000; Liu et al., 2026). Participants respond to each item using a six-point Likert scale and receive a total score which represents the sum of the seven dimensions. The meanings of seven dimensions come as follows (Facione, 1990; Facione et al., 1994, 2000; McBride et al., 2002; Orhan, 2022).

###### 2.1.1.1.1 Truth-seeking

It reflects a disposition characterized by eagerness to seek the best knowledge in any given context, courage to ask questions, and honesty and objectivity in pursuing inquiry-even when the findings contradict one’s self-interests or preconceived opinions.

###### 2.1.1.1.2 Open-mindedness

It assesses someone’s tolerance for divergent views and awareness of potential personal biases.

###### 2.1.1.1.3 Analyticity

It evaluates how one values applying reasoning and evidence to solve problems, anticipates potential conceptual or practical difficulties, and remains consistently vigilant about when intervention is needed.

###### 2.1.1.1.4 Systematicity

It evaluates one’s ability to maintain organization, orderliness, focus, and diligence in inquiry.

###### 2.1.1.1.5 Inquisitiveness

This measures one’s intellectual curiosity and persistent desire for learning, even when the practical application of the knowledge is not immediately clear.

###### 2.1.1.1.6 Self-confidence

This dimension measures the trust individuals place in their own reasoning processes which enables people to trust their sound judgments, and guide others in rational problem-solving.

###### 2.1.1.1.7 Maturity

This assessment targets the disposition toward judicious decision-making. The CT mature individual approaches problems, inquiries, and decisions with the awareness that: some problems are inherently ill-structured, some situations allow multiple plausible solutions, and judgments often require standards, contexts, and evidence that cannot guarantee certainty.

##### 2.1.1.2 Chinese adults version of CCTDI

Several Chinese scholars tried to translate and adapt the English version of CCTDI into a Chinese version of CCTDI, known as CCTDI-CV (Luo and Yang, 2001; Peng et al., 2004; Hou et al., 2022). The CCTDI-CV maintains the same underlying structure and scoring method as with its English version (also see Yeh, 2002; Zeng et al., 2025). The CCTDI-CV has 70 items, including five new items intended to better reflect Chinese students’ perspectives. The five new items are: Item 1: Personal experience is the only criterion that can be used to verify the truth of a statement (for Truth-seeking). Item 2: I do not question what is generally accepted by people (for Open-mindedness). Item 3: I often reflect repeatedly on what is right or wrong in practice and experience (for Systematicity). Item 4: I would give up, if I feel hard to understand a complex product manual (for Systematicity). Item 5: I am afraid to ask questions in class (for Self-confidence).

##### 2.1.1.3 Chinese children version of CCTDI

Some Chinese researchers have modified the CCTDI-CV to make it more accessible for upper elementary and junior middle school students, leading to the development of the Children’s version of CCTDI. Zhi (2021) refined the scale by removing certain items, reducing the total count to 45 items, and rewording complex statements. For instance, the item “In group discussion, if someone’s argument is considered wrong by others, they have no right to express their opinion” from Open-mindedness was rewritten as “If a classmate’s viewpoint is clearly wrong, they have no right to express it, and I am unwilling to try to understand their position” (Zhi, 2021; Lao, 2023).

### 2.2 Search for primary studies

To identify empirical studies on the critical thinking disposition of Chinese students, a systematic search was conducted across several digital databases. The search utilized keywords such as “critical thinking” (or its Chinese equivalents 批判性思维, 审辩, 高阶思维), “scale” (or questionnaire, California Critical Thinking Disposition Inventory), and “Chinese student” (or student), both individually and in combination.

Databases searched included CNKI, Wanfang, Chongq VIP, Taylor & Francis, Sage, Elsevier, John Wiley, Springer, PsycINFO, Education Resources Information Center (ERIC), JSTOR, and ProQuest Dissertations & Theses Global. The search covered documents cataloged before February 7, 2025.

**Figure 1:**
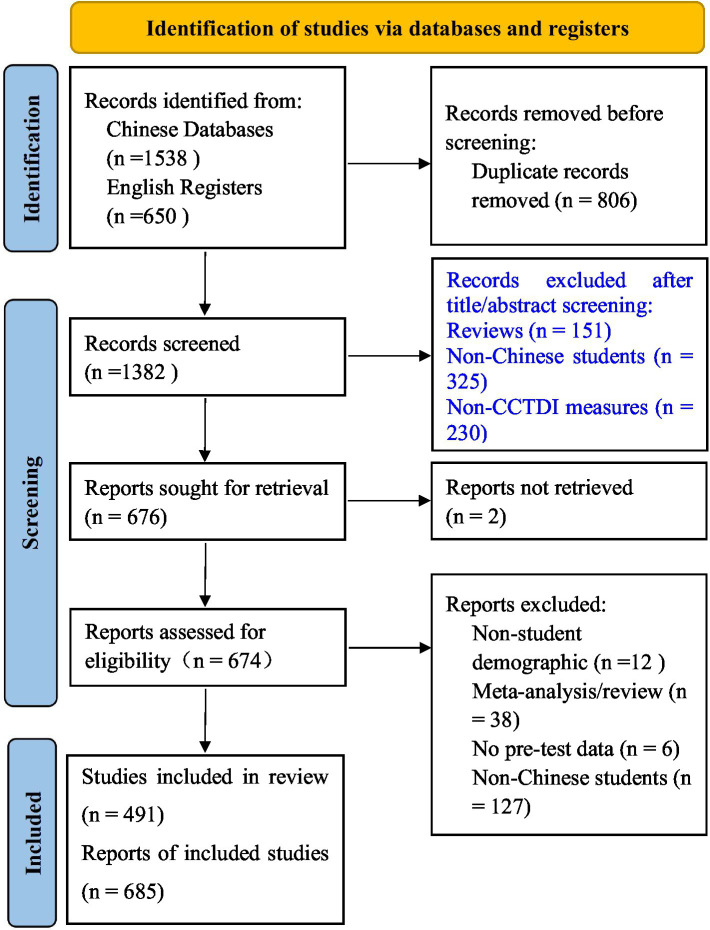
The process of literature searching.

The searching yielded a total of 2,188 primary studies, comprising 1,583 studies published in Chinese and 650 published in English. After full text assessment, 702 studies were selected into the next stage of meta-analysis. The Figure 1 illustrates the systematic search and selection process. The literature search and study selection procedures were conducted and reported in accordance with the Preferred Reporting Items for Systematic Reviews and Meta-Analyses (PRISMA) guidelines (Page et al., 2021; Table 1).

**Table 1:** Database search results.

Database	Search keywords	Number of retrieved articles
CNKI	批判性思维; 审辩; 高阶思维; 量表; 问卷	821
Wanfang	批判性思维; 审辩; 高阶思维; 量表; 问卷	457
Chongq VIP	批判性思维; 审辩; 高阶思维; 量表; 问卷	260
Taylor & Francis	critical thinking; California Critical Thinking Disposition Inventory; student	58
Sage	Critical Thinking; scale; questionnaire; Chinese student	78
Elsevier	California Critical Thinking Disposition Inventory	44
John Wiley	critical thinking; California Critical Thinking Disposition Inventory; Chinese student	279
Springer	critical thinking; California Critical Thinking Disposition Inventory; student	50
PsycINFO	critical thinking; CCTDI; student	44
Education Resources Information Center (ERIC)	critical thinking; California Critical Thinking Disposition Inventory; Chinese student	7
JSTOR	critical thinking; California Critical Thinking Disposition Inventory; student	38
ProQuest Dissertations & Theses Global	critical thinking; California Critical Thinking Disposition Inventory; Chinese student	52

### 2.3 Inclusion and exclusion criteria

To be eligible for inclusion in the current meta-analysis, a primary study must satisfy the following criteria:

- The participants must be Chinese students from the K-12 stage or higher education levels within the Chinese educational system.
- The study must utilize the CCTDI and its variants, including CCTDI-CV, and its Chinese Children version.
- The study must report some essential information, including the sample size, scores for the seven dimensions and total scores of CCTDI (Means and SDs), and other relevant study features (e.g., hometown location, type of university, publication type, see Table 2 and Supplementary Table S1 for details).

**Table 2:** Study features extracted in the primary studies.

Moderators	Primary studies		Independent samples	
	k1	%	k2	%
Hometown locations				
Urban areas	25	54.35%	35	62.50%
Rural areas	21	45.65%	21	37.50%
Grades				
Elementary school	10	2.22%	19	3.02%
Junior middle school	46	10.20%	64	10.16%
Senior high school	176	39.02%	235	37.30%
Undergraduate	193	42.79%	279	44.29%
Postgraduate	26	5.76%	33	5.24%
Type of Major				
Law	2	0.87%	4	1.20%
Engineering	19	8.26%	24	7.19%
Management	5	2.17%	6	1.80%
Education	12	5.22%	18	5.39%
Science	78	33.91%	119	35.63%
Literature	38	16.52%	46	13.77%
Medicine	71	30.87%	106	31.74%
Art	5	2.17%	11	3.29%
Type of university				
Key Universities	44	31.21%	71	31.84%
Non key Universities	97	68.79%	152	68.16%
Type of publication				
General journals	147	30.56%	216	32.05%
Core Journals	58	12.06%	85	12.61%
Dissertations	276	57.38%	373	55.34%

### 2.4 Coding process and coding quality

Coding was conducted by two authors of the article following a structured process. The first step involved developing a coding plan, during which we identified the necessary information to extract from the primary studies. Those study features (i.e., moderators) and other information extracted were listed in Table 3 and Supplementary Table S1. The second step was to extract information from each primary study. The coder GSL initially coded five primary studies and reviewed the coding plan. Finally, a formal coding table was created, and all primary studies were coded accordingly (see Supplementary Table S1 for details). The other coder FHY independently checked the coding table. Any discrepancies or issues were resolved through consultation with a meta-analysis methodological expert.

**Table 3:** The estimated effect sizes and heterogeneity test Results for Chinese students’ CCTDI scores.

CCTDI dimensions	*k*	Mean	SE	95% CI	I^2^	Q
Total Score	674	3.809	0.017	3.777, 3.842	0.995	148388.98***
Truth-seeking	599	3.670	0.016	3.638, 3.702	0.993	91886.48***
Open-mindedness	598	3.909	0.021	3.867, 3.951	0.998	329894.15***
Analyticity	593	3.894	0.024	3.847, 3.94	0.997	181682.64***
Systematicity	593	3.684	0.017	3.652, 3.717	0.994	94812.91***
Self-confidence	598	3.699	0.020	3.661, 3.738	0.990	61578.06***
Inquisitiveness	596	3.957	0.027	3.904, 4.011	0.998	358834.42***
Cognition maturity	592	3.872	0.023	3.826, 3.918	0.996	138991.42***

***Indicates *p* < 0.001.

The coding quality was assessed based on the percentage of correctness and consistency between the two independent coders. The coding accuracy for this project is 100%, whereas consistency between the two coders was 98%.

### 2.5 Statistical computations

#### 2.5.1 Effect size

In this meta-analysis, the mean score served as the effect size indicator (Borenstein et al., 2009; Card, 2012; De Livera et al., 2024). The mean was calculated as the average score of each item. When a primary study reported the total score for a dimension of CCTDI, the mean was derived by dividing the total score by the number of items (i.e., 10 in the current case). For studies reporting a total score of the entire scale, the mean was obtained by dividing the total scale score by the number of items on the scale.

#### 2.5.2 Model selection

The current meta-analysis employed a random effects model. The major difference between a fixed effects model and a random effects model lies in the source of the errors. The fixed effects model assumes that the variance in effect sizes across primary studies is due to random error, and all studies share a common population effect size (Assink and Wibbelink, 2016; Borenstein et al., 2009; Card, 2012; Harrer et al., 2021; Veroniki and McKenzie, 2024). In contrast, the random effects model assumes that the difference in effect sizes among studies arises from both systematic and random errors (Assink and Wibbelink, 2016; Borenstein et al., 2009; Card, 2012; Harrer et al., 2021; McKenzie and Veroniki, 2024). The choice of models here is based on the purpose of research. While the fixed effects model does not permit generalization of the findings to a broader population, the random effects model allows generalization to a population (Assink and Wibbelink, 2016; Harrer et al., 2021; McKenzie and Veroniki, 2024).

#### 2.5.3 Homogeneity test

In this meta-analytic study, the *Q* test and *I*^2^ were used to assess the homogeneity of the primary studies. If the *p*-value from the *Q* test is less than 0.050 (at the alpha = 0.05), this suggests that the effect sizes among the studies are heterogeneous; otherwise they are considered as homogeneous (Borenstein et al., 2009; Card, 2012; Harrer et al., 2021; Higgins et al., 2024, Chapter 10). The *I*^2^ statistic was used to describe the extent of between-study heterogeneity. Because interpreting *I*^2^ using fixed thresholds can be misleading, this study followed commonly used ranges rather than strict cut-offs. In general, *I*^2^ values of approximately 0–40% may indicate heterogeneity that is not important; 30–60% may represent moderate heterogeneity; 50–90% may represent substantial heterogeneity; and 75–100% may indicate considerable heterogeneity (Higgins and López-López, 2025; Higgins et al., 2024, Chapter 10).

#### 2.5.4 Detection of publication biases

Publication bias refers to the tendency for studies with statistically significant findings to be more readily accepted for publication, while studies with non-significant findings may face greater difficulty in being accepted for publication (Borenstein et al., 2009; Card, 2012; Harrer et al., 2021; Higgins et al., 2024, Chapter 7). This meta-analysis examined the potential publication bias among the included primary studies. The fail-safe N (Nfs) is defined as the number of studies that would need to be added into the pool of primary studies for the overall results to lose their significant difference (Borenstein et al., 2009; Card, 2012; Harrer et al., 2021; Afreixo et al., 2026). A critical Nfs value of 5 k + 10 is used. If Nfs exceeds this value, publication bias is detected; otherwise, no publication bias is detected (Borenstein et al., 2009; Card, 2012; Harrer et al., 2021; Afreixo et al., 2026).

#### 2.5.5 Assessment of quality

The quality of the primary studies included in this meta-analysis was assessed using the Basic Quality Assessment of Primary Study (BQAPS) (Xu et al., 2024), which was developed based on the Cochrane Risk of Bias framework (Higgins et al., 2024, Chapter 8). The BQAPS evaluates core methodological aspects of primary studies, including research design, measurement reliability and validity, statistical methods, and implementation procedures.

The BQAPS consists of 12 items, each rated on a three-point ordinal scale: 0 (insufficient information for judgment), 1 (does not meet methodological requirements), and 2 (adequate information and meets methodological requirements). Higher scores indicate lower risk of bias. The total BQAPS score ranges from 0 to 24 and was used both for categorical classification and as a continuous indicator of study quality.

Based on the total scores, studies were categorized as low quality (0–6), low-medium quality (7–12), medium-high quality (13–18), and high quality (19–24). To enhance transparency and reproducibility, the detailed BQAPS criteria and a brief example of the scoring procedure are provided in the Supplementary materials.

To enhance transparency and reproducibility, a detailed description of the BQAPS criteria and an example of item-level scoring are provided in the Supplementary materials (see also Xu et al., 2024; full instrument available at: https://osf.io/u3syb/?view_only=c8894b14dd28406da57257bd9930a010).

#### 2.5.6 Statistical calculation tools

All statistical analyses were conducted using R 4.4.3 (R Core Team, 2023). The meta package was used to compute the random effects model and create the funnel plot (Schwarzer, 2025; Schwarzer et al., 2015), and the Metafor package was used to compute the Nfs (Balduzzi et al., 2019; Viechtbauer, 2010, 2025).

## 3 Results

### 3.1 Characteristics of primary studies included

Out of 702 primary studies retrieved, 491 studies met the criteria mentioned above (see Table 2; Supplementary Table S1), comprising 688 independent samples and 146,625 participants. These studies were published between 2000 and 2025.

The meta-analytic sample is both diverse and representative (see Table 2). In terms of geographic distribution, individual studies included 35 from urban areas (*N* = 13,409), and 21 from rural areas (*N* = 6,514).

These primary studies encompass all grade level. Specifically, the numbers of studies for elementary schools, junior middle schools, senior middle schools, undergraduates, and postgraduates were 19 (*N* = 6,623), 64 (*N* = 9,374), 235 (*N* = 42,070), 279 (*N* = 69,136), and 33 (*N* = 8,402), respectively.

These individual studies also originated from different types of university. Specifically, 71 (*N* = 10,907) were from key universities, whereas 152 (*N* = 28,721) were from non-key universities.

### 3.2 Results of heterogeneity tests

The *Q*-test value for the CCTDI total score is 148388.980, *p* < 0.001, while the Q-test values for every dimension of CCTDI range from 61578.060 to 358834.420, all *p*s < 0.001. The *I*^2^ for the CCTDI total score is 99.50%, with *I*^2^ value for its dimensions of CCTDI ranging from 99.00 and 99.80%.

These values (Table 3) indicate substantial heterogeneity in the effect sizes for the total score and individual dimensions of CCTDI, underscoring the need for moderator analyses to examine the potential moderating effects of relevant variables.

### 3.3 Results of publication tests

**Figure 2:**
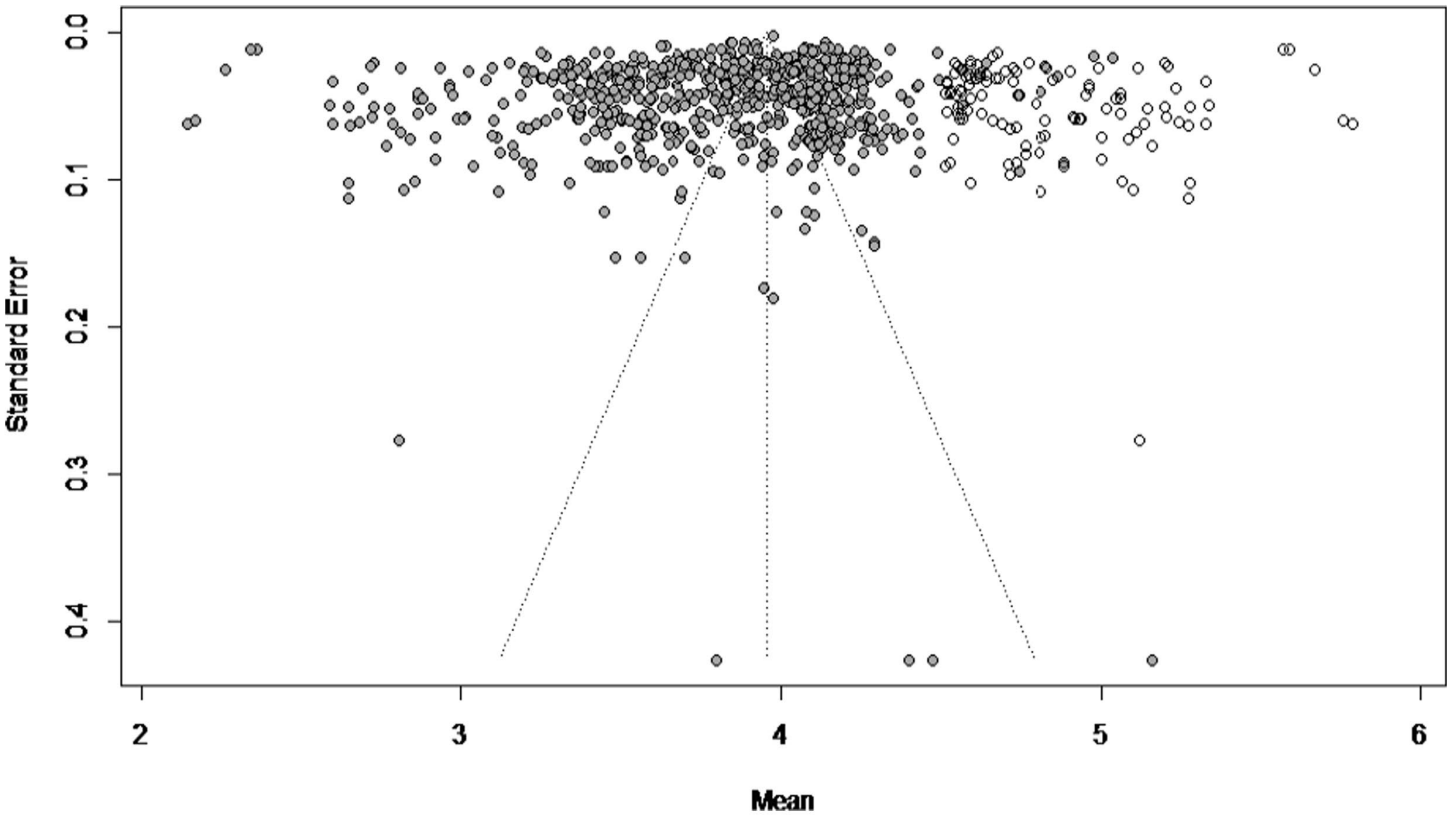
The trimmed funnel plot of the current meta analysis.

The funnel plot analysis indicated an asymmetrical distribution of effect sizes (see Figure 2), showing that missing effect sizes were primarily on the right. The trim-and-fill method estimated 57 missing effect sizes. After imputing these values, the re-estimated effect size was 3.954 (95% CI [3.915, 3.993]), closely aligning with the pre estimated effect size of 3.809 (95% CI [3.777, 3.824]).

Rosenthal’s fail-safe N is 10,000,000, which far exceeds the critical threshold of 3,450 (calculated as 5 *k* + 10 = 5 × 688 + 10; Card, 2012).

According to established guidelines (Borenstein et al., 2009; Card, 2012; Harrer et al., 2021; Higgins et al., 2024), funnel plot asymmetry can suggest small-study effects or selective reporting, as publication bias typically arises because journals tend to favor studies reporting larger or statistically significant effects (Rosenthal, 1979; Borenstein et al., 2009; Card, 2012; Harrer et al., 2021; Higgins et al., 2024, Chapter 13). However, in the present meta-analysis, the effect sizes are generally concentrated at relatively low values. Therefore, the observed asymmetry may not solely reflect publication bias, but could also partially represent the underlying distribution of critical thinking disposition scores among Chinese students. Overall, these findings should be interpreted cautiously, acknowledging the potential for minor bias while recognizing that the true distribution may contribute to the pattern.

### 3.4 The impact of quality of primary studies

Among the 491 primary studies, none fell into the Medium-Low Quality category (quality assessment scores: 7–12). A total of 211 studies (42.970%) were classified as Medium-High Quality (scores: 13–17), whereas 280 studies (57.030%) were classified as High Quality (scores: 18–24).

The meta-regression analysis indicated that no significant relationship between quality scores and the total score of Chinese students’ critical thinking disposition. The meta-regression equation did not reach the statistical significance, *F* (1, 672) = 3.528, *p* = 0.061, intercept = 3.457, *SE* = 0.189, *t*(672) = 18.332, *p* < 0.0001.

### 3.5 Main effect

As shown in Table 3, the estimated total effect size (ES) for Chinese students’ critical thinking disposition is 3.809 (*k* = 674, 95%*CI* [3.777, 3.842]), representing 63.48% of the 6-point Likert scale.

The estimated ES for Chinese students’ Truth-seeking is 3.670 (*k* = 599, 95% *CI* [3.638, 3.702]), accounting for 61.170% of the 6-point Likert scale. The estimated ES for Open-mindedness is 3.909 (*k* = 598, 95%*CI* [3.867, 3.951]), representing 65.150% of the 6-point Likert scale. The estimated ES for Analyticity is 3.894 (*k* = 593, 95%*CI* [3.847, 3.940]), accounting for 64.900% of the 6-point Likert scale. The estimated ES for Systematicity is 3.684 (*k* = 593, 95%*CI* [3.652, 3.717]), which accounted for 61.400% of the 6-point Likert scale.

The estimated ES for Chinese students’ Self-confidence is 3.699 (*k* = 598, 95%*CI* [3.661, 3.738]), accounting for 61.650% of the 6-point Likert scale. The estimated ES for Inquisitiveness is 3.957 (*k* = 596, 95%*CI* [3.904, 4.011]), representing 65.960% of the 6-point Likert scale. The estimated ES for Cognition Maturity is 3.872 (*k* = 592, 95%*CI* [3.826, 3.918]), accounting for 65.5% of the 6-point Likert scale.

### 3.6 Results of moderator analyses

#### 3.6.1 Effect of hometown location

Based on the level of economy development, the primary studies were classified into two subgroups: the urban groups (*k* = 35) and the rural groups (*k* = 21). The results (*Q*_between_ = 0.170, *p* = 0.677) indicated that there was no significant difference among these subgroups.

#### 3.6.2 Effect of grades

According to the reported grade information, those primary studies were classified into five subgroups: the elementary school group (*k* = 19), the junior middle school group (*k* = 64), the senior middle school group (*k* = 235), the general university student group (*k* = 279), the postgraduate student group (*k* = 33).

**Figure 3:**
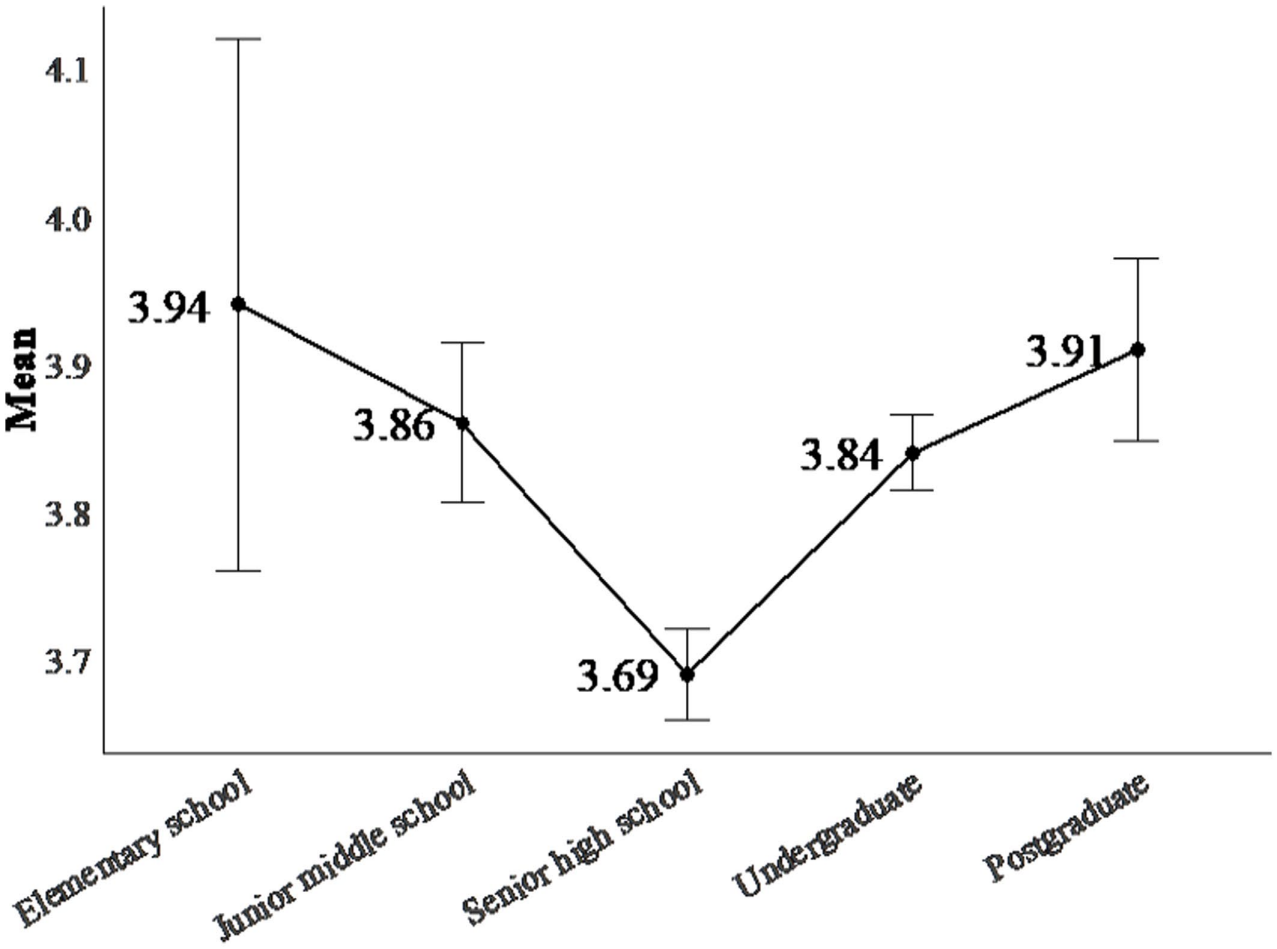
The grade effect in Chinese students’ critical disposition.

The mean differences among these five subgroups deviated significantly from what would be expected by random error, *Q*_between_ = 19.460, *p* < 0.001. The trajectory of Chinese students’ critical thinking disposition follows a V-shaped curve (see Figure 3). In the first half, from the elementary school stage to the senior high school stage, there is a downward trend, with a decline from *ES* = 3.939 to *ES* = 3.693. In the second half, from senior high school to the postgraduate levels, there is an upward trend, with *ES* increasing from *M* = 3.693 to *M* = 3.910.

#### 3.6.3 Effect of major type

According to the reported major description, the primary studies were divided into eight subgroups: the Law group (*k* = 4), the engineering group (*k* = 24), the Management group (*k* = 6), the Education group (*k* = 18), the Science group (*k* = 119), the liberal arts group (*k* = 46), the Medicine group (*k* = 106) and the Arts (*k* = 11).

The results of mean difference test indicated that the mean differences among these eight subgroups did exceed the range due to random error effect, *Q*_between_ = 14.77, *p* = 0.039. The ranking order of ES from the lowest to the highest is as follows. The Science major group (*M* = 3.778) < the Engineering major group (*M* = 3.785) < the Management major group (*M* = 3.828) < the Medicine major group (*M* = 3.867) < the Law major group (*M* = 3.873) < the Art major group (*M* = 3.893) < the Education major group (*M* = 3.967) < the Literature major group (*M* = 3.980).

#### 3.6.4 Effect of university type

Based on the reported university information, the primary studies were divided into two subgroups: the non-key university (*k* = 152) and the key university (*k* = 71). The Q test results indicated a significant mean difference between these two types of universities, *Q*_between_ = 25.23, *p* < 0.001. Students from key universities (*M* = 4.025, *SE* = 0.037) had higher critical thinking disposition scores than those came from non-key universities (*M* = 3.769, *SE* = 0.036) (See Table 4 for details).

**Table 4:** Results of moderator analyses.

Moderators	*k*	Mean	SE	95%CI	Q_b_
Hometown locations					0.17
City areas	35	4.117	0.081	3.958, 4.276	
Rural areas	21	4.065	0.102	3.866, 4.265	
Grades					19.46***
Elementary school	19	3.939	0.180	3.587, 4.291	
Junior middle school	64	3.860	0.054	3.754, 3.967	
Senior high school	235	3.693	0.031	3.632, 3.755	
Undergraduate	279	3.842	0.026	3.792, 3.892	
Postgraduate	33	3.906	0.062	3.785, 4.027	
Type of Major					14.77*
Law	4	3.873	0.256	3.37, 4.375	
Engineering	24	3.785	0.090	3.608, 3.962	
Management	6	3.828	0.164	3.506, 4.149	
Education	18	3.967	0.066	3.837, 4.096	
Science	119	3.778	0.045	3.689, 3.866	
Literature	46	3.980	0.042	3.897, 4.063	
Medicine	106	3.867	0.032	3.804, 3.929	
Art	11	3.893	0.085	3.726, 4.061	
Type of university					25.23***
Key Universities	71	4.025	0.037	3.952, 4.097	
Non key Universities	152	3.769	0.036	3.699, 3.84	
Type of publication					16.17***
General journal	216	3.876	0.024	3.828, 3.924	
Core Journals	85	3.895	0.044	3.808, 3.982	
Dissertation	373	3.751	0.024	3.703, 3.799	

*Indicates *p* < 0.05, ***indicates *p* < 0.001.

#### 3.6.5 Effect of publication type

According to the reported publication information, the primary studies were categorized into three subgroups: the general public journal group (*k* = 216), the core journal group (*k* = 85), and the dissertation group (*k* = 373). The Q test results indicated significant mean differences among these three subgroups, *Q*_between_ = 16.17, *p* < 0.001. Specifically, the effect sizes for the dissertation group, general journal group, and core journals group are 3.751, 3.876, and 3.895, respectively.

## 4 Discussion

To clarify its contribution in relation to prior reviews, particularly Fan and See (2022) and Jiang et al. (2024), the present study extends the literature in several important ways. Unlike previous reviews that relied on narrative synthesis or vote-counting approaches, this study employs meta-analytic techniques to estimate pooled mean scores of Chinese students’ critical thinking disposition, thereby providing a quantitative approximation of population-level parameters. Moreover, it synthesizes an unprecedented dataset of 491 primary studies, 688 independent samples, and over 146,000 participants. Through moderator analyses, the study systematically examines sources of heterogeneity across educational stages, disciplinary fields, institutional types, and publication types-analyses that were not feasible in earlier reviews. Finally, the findings are interpreted within a human capital and ecological framework, offering a theoretically grounded explanation for variations in critical thinking disposition across educational contexts.

### 4.1 Pooled scores for critical thinking disposition of Chinese students

The primary and most significant finding of the current study is the meta analyzed data for critical thinking disposition score of Chinese students (see Table 3). The averaged total critical thinking disposition score of Chinese students is 3.809 (*k* = 494, 95% *CI* [3.777, 3.842]) on a 6-point Likert scale. The scores of seven subscales range from 3.670 to 3.909.

The single pooled estimates can help us to capture the central tendency of Chinese students’ critical thinking disposition. However, it should be noted that these single pooled estimates are computed based on a dataset with extreme heterogeneity. Therefore, subgroup-based estimates (in Table 4) are better suited for explaining individual scores on CCTDI.

### 4.2 Significant effect of grade

The second key finding is the significant moderating effect of grade, which reveals a V-shaped pattern in the development of Chinese students’ critical thinking disposition from elementary school to postgraduate level (as shown in Figure 3). Within this pattern, senior high school students exhibit the lowest critical thinking disposition scores.

The current finding expands on previous studies by covering the full range of grades, from elementary to postgraduate, while previous research has focused on specific periods within this range (e.g., Qi and Sun, 2020; Shen et al., 2019; Yu, 2023).

The V-shaped trend of the grade effect is supported by several studies (e.g., Qi and Sun, 2020; Zhang et al., 2009; Chen et al., 2024). This significant grade effect may be associated with several factors, including natural cognitive development, repetitive exercise and homework, and opportunities to practice critical thinking.

First, the downward trend during the K-12 stage, in contrast to the cognitive development patterns (Park et al., 2023; Qiang et al., 2020; Said-Metwaly et al., 2021; Woolfolk, 2013), may be due to excessive repetitive exercise and homework. Both K-12 teachers and students primarily focus on improving exam scores through rote exercise, which may hinder the development of Chinese students’ critical thinking disposition (Zheng, 2013; Xie et al., 2024). Meta-analytic evidence indicates that average daily homework time increased from 32.36 to 38.66 min in elementary school, from 42.18 to 48.54 min in junior middle school, and from 46.79 to 63.63 min in senior high school between 2004 and 2020 (Zhai and Fan, 2021), potentially contributing to lower scores in senior high school.

Second, the upward trend in the latter half of the V-shaped curve could be related to increasing opportunities for critical thinking exercises. After the senior high school stage, these opportunities expand significantly. At the university level, two required courses – *research methods* and *dissertation* – offer students the initial training in critical thinking (The Teaching Steering Committee of Higher Education Institutions of the Ministry of Education of China, 2018). At the postgraduate level, students are required to take at least one compulsory course, such as research methods, which explicitly teaches the systematic application of critical thinking. This structured training may offer valuable opportunities to practice these skills under higher academic standards and expectations (China National Guidance Committee of Professional Postgraduate Education, 2020). In contrast, no such courses are available during the K-12 stage, leaving students with extremely limited opportunity to develop critical thinking skills (China Ministry of Education, 2022a, 2022b, 2022c, 2022d).

### 4.3 Significant effect of university type and insignificant effect of hometown location

The significant effect of grade can be explained by human capital theory (Blair, 2011; Brown, 2006; Fang, 2021; Leoni, 2025; Sycheva et al., 2019). Financial support increases progressively from lower grade to higher grade. In 2023, the financial investments per student were 12,529.740 Yuan for elementary school, 17331.380 Yuan for junior middle school, 18092.820 Yuan for senior middle school, and 21260.460 Yuan for university (China Ministry of Education, National Bureau of Statistics, Ministry of Finance, 2024). These figures indicate that university students received nearly twice the financial support of elementary students. The grades receiving higher financial support may provide greater resources and opportunities to foster critical thinking disposition among Chinese students.

Similarly, the significant effect of university type is consistent with the predictions of human capital theory (Blair, 2011; Brown, 2006; Fang, 2021; Leoni, 2025; Sycheva et al., 2019). Empirically, key universities in China receive substantially greater financial support than non-key universities (Zhou, 2023; Longhuyunye, 2023). These higher investments contribute to a higher quality of teaching and an improved learning environment, which may facilitate more effective critical thinking exercises and offer greater opportunities to develop critical thinking dispositions (Blair, 2011; Brown, 2006; Fang, 2021; Leoni, 2025; Sycheva et al., 2019). Chen and Chen (2016) reported that Sichuan university allocated substantial financial resources to reduce the size of public math classes, which led to significant improvements in students’ math scores.

Concerning the insignificant effect of hometown location, it is consistent with the negative predictions of human capital theory (Blair, 2011; Brown, 2006; Fang, 2021; Leoni, 2025; Sycheva et al., 2019). First, financial investment differences across urban and rural areas are minimal (China Ministry of Education, National Bureau of Statistics, Ministry of Finance, 2024). Chinese government has rigorous policy to reduce the financial differences between urban and rural areas since several decades ago. Second, there is a high degree of educational homogeneity. Both urban and rural areas in China follow the same curriculum standards, use identical textbooks, and have similar teacher training requirements. This homogeneity may contribute to limited variations in critical thinking disposition scores.

### 4.4 Significant effects of publications and majors

The present study found a significant moderating effect of publication type, consistent with H6. This pattern is supported by methodological literature (Borenstein et al., 2009; Card, 2012; Harrer et al., 2021; Higgins et al., 2024, Chapter 7), which suggests that the core journal has tendency to favor significant results. However, as indicated in Figure 2, this bias can be disregarded.

The present study also found a significant moderating effect of major. Notably, the results show a trend opposite to previous research (Ma et al., 2015; Amelink et al., 2024), indicating that humanities majors exhibit a higher critical thinking disposition than STEM majors. In Chinese culture, humanities researchers may be perceived as having stronger spirit of questioning and criticism than STEM counterparts (Lei and Liu, 2018; Wang and Shen, 2022). For instance, sociologist Zheng (2013) is a prominent figure known for criticizing educational alienation. Conversely, there has been no notable instance of a STEM scholar publicly addressing this issue in China. Similarly, Wang and Shen (2022) found that female STEM students show significantly lower gains in critical thinking ability during college compared to female humanities students, highlighting the role of disciplinary context in shaping critical thinking development.

Another plausible explanation is that this result may be partly explained by the higher self-confidence of humanities students rather than their actual utilization of critical thinking skills. Humanities students are frequently encouraged to explore complex issues, consider diverse perspectives, challenge assumptions, and defend their arguments, which may foster greater confidence in critical thinking. This contrasts with STEM students, who tend to focus on problem-solving and established principles, with less emphasis on open-ended inquiry. This makes critical thinking appear less central in STEM disciplines. As a result, humanities students may feel more confident in their critical thinking abilities because of the focus on critique, debate, and reflection, along with greater exposure to and engagement in social and cultural topics, which may promote their self-perception of critical thinking. Given that our main findings indicate a generally low level of critical thinking disposition among Chinese students (see Table 3), this interpretation warrants further investigation.

### 4.5 The theoretical implications

From a theoretical perspective, the following two points are worth elaborating on deeply. First, the findings of this study extended the previous primary studies by a meta analyzed framework in which characteristics of primary studies and effect sizes were correlated. Besides estimating the true level of critical thinking disposition among Chinese students, this study also identified characteristics of the original studies—such as grade level, academic discipline, and institution type—that were significantly related to the variation in effect sizes. These two points did not appear in all previous primary studies.

Second, the current meta analysis applied the human capital theory (Blair, 2011; Brown, 2006; Fang, 2021; Leoni, 2025; Sycheva et al., 2019) to coherently explain the variances among effect sizes within grade, type of major, type of university. The current work identified a specific social factor (i.e., financial support) which may be related to the environmental influences proposed by Bronfenbrenner’s ecological model (Bronfenbrenner, 1977; Bronfenbrenner and Morris, 2007; Woolfolk, 2013; Tong and An, 2024) and the students’ development of critical thinking dispositions. Our work here extended Bronfenbrenner’s work because Bronfenbrenner’s early theory broadly proposed environmental influences on development without specifying any specific factor.

Furthermore, the current work puts forth theoretical issues unknown. How does financial support influence students’ critical thinking dispositions via curriculum standards, instructional processes, and learning impacts? Does teachers’ perception of curriculum standards serve as a mediating factor between funding allocation, instructional practices, student learning, and critical thinking dispositions? These questions deserve further examination.

### 4.6 The educational implications

The findings of the current meta analysis carry important educational implications. First, these pooled scores can be used as a reference criterion to explain the CCTDI test scores of Chinese students in future. If the new CCTDI test takers are ‌purely homogeneous groups‌ (e.g., exclusively primary school students, junior secondary students, senior high school students, or university students), reference criterion may be selected from ‌Table 4 for a specific subgroup‌. When the new CCTDI test takers comprise ‌mixed grades‌, reference criterion‌ can be found in ‌Table 3. It should be noted that ‌this approach is provisional‌, applicable ‌only pending the development of an indigenous Chinese version of the critical thinking disposition scale‌.

Second, the observed “V-shaped effect” across grade levels suggest that repetitive, excessive, and monotonous learning experiences in K-12 stage may impede the development of critical thinking. To address this issue, fostering critical thinking should be a core objective in the Chinese education system. Introducing mandatory courses such as *Introduction to Logic* and *Introduction to Critical Thinking* could potentially help bridge this gap in the Chinese education system, whereas existing courses like *The Fundamentals of Marxism* (Zhang, 2017; Zhuo, 2024) and *Research Methods* should integrate critical thinking training to better equip students with higher critical thinking disposition.

### 4.7 Potential sources of heterogeneity

The present study found that I^2^ values for the CCTDI total score and all its subscales exceeded 99%, indicating extremely high heterogeneity among the included studies. Such high heterogeneity does not undermine the validity of the findings; rather, it reflects the diversity of the included studies in terms of sample characteristics, educational stages, disciplinary fields, CCTDI versions, and regional contexts. Specifically, potential sources of heterogeneity include:

- *Differences in CCTDI versions*: This study included the original English version, the Chinese Adult version (CCTDI-CV), and the Children’s version. Variations in item wording, number of items, and cultural adaptation may have contributed to differences in effect sizes.
- *Grade-level differences*: Moderator analyses indicated a V-shaped developmental pattern in Chinese students’ critical thinking disposition. The integration of samples from different educational stages increases overall heterogeneity.
- *Socioeconomic and regional contexts*: Although no significant urban–rural differences were observed, school resources, financial investment, and regional development levels may influence students’ critical thinking disposition, adding to inter-study variability.
- *Disciplinary fields and university types*: Students from different majors and university types showed significant differences in critical thinking disposition, representing an important source of effect size variation.
- *Data collection procedures and study quality*: Included studies varied in sampling methods, survey administration, and quality scores. Although most studies were of medium-to-high quality, methodological differences could still lead to fluctuations in effect sizes.

In summary, the high I^2^ values reflect the complexity and diversity of research on Chinese students’ critical thinking disposition. Through our moderator analyses, we have identified several key sources of heterogeneity, including grade level, major, university type, and publication type, providing a theoretical basis for understanding differences in critical thinking development across educational contexts.

It is important to emphasize the methodological implications of the exceptionally high heterogeneity observed in this meta-analysis. I^2^ values exceeding 99% indicate extreme between-study variability, suggesting that the included studies differ substantially in their populations, educational contexts, measurement conditions, and institutional settings. Under such circumstances, the pooled mean estimate should not be interpreted as a precise or stable description of any specific student group. Rather, it represents a broad indicator of central tendency across highly diverse contexts, derived from a random-effects model that explicitly allows true effects to vary across studies. This level of heterogeneity necessarily limits the stability and practical precision of a single pooled estimate when treated as a reference value. Accordingly, the pooled mean is best understood as a general reference for contextual comparison rather than as a normative or operational benchmark applicable to individual institutions, regions, or educational stages.

To partially address this limitation, moderator analyses were conducted to examine whether heterogeneity could be reduced within more homogeneous subgroups. As shown in Table 4, certain moderator-defined categories, such as grade level, university type, and publication type-exhibited comparatively narrower confidence intervals than the overall pooled estimate, suggesting relatively greater internal consistency within these subsets. For example, senior high school students (*k* = 235) and undergraduate students (*k* = 279) showed relatively tight confidence intervals, reflecting greater stability in effect size estimates within these educational stages. Similarly, subgroups with larger numbers of studies, such as science and medicine majors, as well as key and non-key universities, demonstrated more precise estimates than smaller subgroups (e.g., law, management, or art majors).

Nevertheless, even within these moderator-defined subgroups, heterogeneity remained substantial, indicating that moderator variables could explain only part of the observed variability. Therefore, while subgroup-specific estimates may offer more context-sensitive reference values than the overall pooled mean, all findings should be interpreted with appropriate caution, particularly when extrapolated to specific institutions, regions, or narrowly defined student populations. These observations underscore the importance of considering heterogeneity when interpreting meta-analytic results and using pooled estimates as reference values.

### 4.8 Consideration of publication bias

Rosenthal’s fail-safe N indicates that the meta-analysis is robust to potential unpublished null results. Nevertheless, the funnel plot revealed some asymmetry, with missing effect sizes primarily on the right. The trim-and-fill procedure suggested 57 potentially missing effect sizes, which slightly increased the pooled estimate from 3.809 to 3.954.

This asymmetry may be due to small-study effects or selective reporting, but it may also partly reflect the underlying distribution of critical thinking disposition scores. Importantly, the overall change after imputation is small, suggesting that the main conclusions remain generally stable. Therefore, the results should be interpreted with caution, acknowledging the possibility of minor bias, without assuming that publication bias is the sole explanation for the observed pattern.

### 4.9 Limitations and issues for further investigation

Although this study reveals several interesting results, it also has several limitations.

Firstly, the meta-analysis did not include all critical thinking dispositions of Chinese students. The CCTDI originates from American culture and fails to encompass all aspects of critical thinking dispositions in Chinese culture. For example, ethical dimensions, which are particularly emphasized in Chinese culture (Xie et al., 2025), are not included in the CCTDI.

Secondly, the meta-analysis included studies using different versions of the CCTDI, including the original English version, the Chinese Adult version, and the Children’s version. The pooled estimates therefore implicitly assume measurement comparability across these instruments. However, this assumption cannot be fully guaranteed due to potential differences in item wording, number of items, and cultural adaptation. Readers should bear this limitation in mind when interpreting the findings.

Thirdly, the number of primary studies in some subgroups is quite small. Specifically, for the type of major, the number of primary studies is limited for certain majors, including Law (*k* = 4), Management (*k* = 6), and Art (*k* = 11).

To advance research on critical thinking disposition, future studies should take the following steps. First, given the critical thinking is an independent construct (Lombardi, 2023), a new indigenous scale measuring the Chinese critical thinking disposition should be developed. Such a scale has been developed (e.g., Critical Thinking Disposition Questionnaire for Primary and Secondary School Students in China, Chen et al., 2024), but they still ignore some connotations valued in Chinese culture (Xie et al., 2025), like CCTDI. To reflect the Chinese psychological content, constructing a new scale would be beneficial to serve as a foundation for further investigation.

Second, more studies should be conducted to examine the critical thinking disposition of Chinese elementary and junior middle school students, as well as students majoring in Law, Management, and Art at higher education.

Third, future research should investigate the mechanistic pathways through which financial support influences critical thinking dispositions via curriculum standards, instructional processes, and learning progression. How does the teachers’ understanding of curriculum standards affect the cultivation of students’ critical thinking dispositions? Which dimensions of instructional behaviors actively inhibit the development of critical thinking dispositions? What qualitative features of student learning behaviors constitute impediments to critical thinking dispositions?‌.

## 5 Conclusion

To accurately assess the critical thinking dispositions of Chinese students and identify potential factors contributing to such inconsistencies in the literature, this study conducted a meta-analysis of 688 independent samples, involving 146,625 participants. Our analysis found that (a) the critical thinking disposition scores of Chinese students decreased from elementary school (mean = 3.939, SE = 0.180) to senior high school (mean = 3.693, SE = 0.031), and recovered in higher education stage (mean = 3.842, SE = 0.026), and (b) factors such as grade level, major type, and university type significantly contributed to variances in effect sizes. Given that this is the first meta analysis, to our knowledge, that has quantitatively synthesized Chinese students’ critical thinking disposition studies, there is a critical need to pursue this line of investigation further.

## Data Availability

The raw data supporting the conclusions of this article will be made available by the authors, without undue reservation.
